# Reports of Case

**Published:** 1857-08

**Authors:** D. L. M’Gugin

**Affiliations:** Prof. of Physiology, Pathology, &c., College of Physicians and Surgeons, Iowa University


### Report of Cases.

BY D. L. M'GUGIN, A. M., M. D.,

(Prof. of Physiology, Pathology, &c., College of Physicians and Surgeons, Iowa University.)

### *Veratrum Viride.*

Since making up my last report of my experience with the above agent, and before it went to press, several other cases of pneumonitis fell under my care in common with my partner, I. P. Letcher, M. D., who had recently undertaken to share my labors with me. He, like myself, had not previously to this, had any experience in the use of the remedy, but after witnessing its effects, was constrained to give it a very prominent place in the list of medical agents. After witnessing its powers in several cases of pneumonitis we were required to prescribe for an adult lady, laboring under measles, in which there was more than usual pulmonary disturbance. This was manifestly acute bronchitis, with some general congestion of the lungs. The pyrexial symptoms ran high, pulse hard and frequent, skin hot; cough dry, distressing, and attended with strong expiratory effort. This state of the case continued for the period of four or five days and yet there was no eruption.

Febrifuges with all the remedies resorted to for the reduction of the heat of skin and the pulse having failed, recourse was had to the veratrum which very soon accomplished what was not effected by other measures, and very soon the eruption appeared on the surface. In due time there was a complete recovery.

In other cases occurring of this sthenic character, and of other types of disease, it was resorted to, universally exhibiting its sedative control, (these words better express the idea than any others which we can use,) for in every case, where the article was pure, it seemed to exercise an irresistible power over the heart and arteries, as if to say, "thus far shalt thou go and no farther." Later in the season two cases presented themselves in an acute form with the usual severe and harrassing symptoms, which it is unnecessary now to detail because they are familiar to every practitioner of experience.

The first was that of a lady in which colchicum, nitras potassæ, opium and calomel were used. Ten days elapsed, but little or no relief followed, and yet with these remedies the treatment was heroic. The pulse continued firm and frequent, the skin dry, the joints inflamed, swollen and painful. The veratrum was then given; the pulse became slow,

Rheumatism the skin covered with a copious perspiration, the pain alleviated to an almost entire exemption, the urine depositing the urate of amonia, the bowels freely opened, the tongue clean, and the movements of limbs and body effected without the agonizing suffering which she had endured previous to the exhibition of the veratrum. The case went forward in speedy convalescence.

The next was a resident of Virginia on a temporary visit to this place, a gentlemen of plethoric habit, and had several times been similarly affected. He was unable to turn in his bed, and when aid was afforded for that purpose, the movement was accomplished with much suffering. His back, his shoulders, arms, his lower limbs were the seats of much suffering, not in the joints only, but along the course of the muscles. The wrists were much swollen, but were not more the source of suffering than parts which were not tumefied. The tongue was loaded and furred, and there was fever, restlessness, scanty urine, and torpid bowels.

Calomel, opium, colchicum, and the usual remedies in such cases were used in a heroic manner, and yet the case threatened to outlive the allotted term of Dr. Watson, of "six weeks duration."

The veratrum was appealed to, but for the first time under my observation did it fail to make a perceptible change in the pulse, and yet Dr. Letcher had ordered it in ordinary doses. We were disappointed, but upon inquiry as to the purity of the article, we were led to suspect that the failure was ascribable to an inferior, or an inert article, and a change to that which we knew to be genuine, proved our suspicions to have been correct. The pulse soon gave evidence of its powers; the skin became relaxed, and the bowels poured out largely of highly offensive matter. He continued to improve, and says his disease is "broken up."

In this case there was a plethoric diathesis in which digestion and nutritive assimilation were performed with activity and efficiency. Not only was there a hyperæmic condition of the blood, but there was also a changed condition of the properties of the circulating fluid, for there is, in such cases, an excess of fibrin. If fibrin is nothing more than a higher condition of the albumen, and rendered so through the agency of oxygen, then the fibrin is, therefore, the albumen oxydized. But fibrin is the oxydizable principle and in these polyœmic habits, more is produced than is required or than can be used. It is, therefore, retained in the blood, and its presence tends to an inflammatory state, because it is highly vitalized. Pyrexia will follow, the secretions become impaired, the nervous system excited, because highly endowed through the agency of blood which is too stimulating and exciting. Andral says: "I have already established the fact that, under the influence of a state of general hyperæmia, every organ becomes excited," and again he says, when speaking of a morbid element generated in the blood by particular influences, that this change is produced by "an abnormal chemical action of the blood itself." Under this state of things it is not to be wondered at that we should have local inflammation of a part through which the blood flows at least not too freely. Congestions would take place, and the concomitants of inflammation—heat, redness, swelling and pain—would follow. Nor is the metastasis from one locality to another a source of wonder when the nervous system is in a condition to favor the establishment of a series of morbid sympathies, for the eratic habits of these local disturbances go to prove the neuropathic character of the inflammation, accompanying the arthrodeal forms of this disease.

It is not my purpose now to follow up the discussion as to the presence of the acids in the blood. This doubtless is the result of a continuance of the morbid states found in the acute—these manifesting themselves in the chronic forms—but I desired more especially to call the attention of the reader to a few prominent pathological conditions in order to account for the benefits arising from an arterial sedative in such cases. Not only is the heart subdued in its excesses, but the respiration becomes normal, and as the excretions are awakened, the morbid materials are from time to time separated away. But the heart must be held down under control by it until the secretions are improved, for if the heart be allowed to drive into the lungs more blood than can be aerated, dyspnea will be the result, because accumulations have taken place in the lungs, which being filled to engorgement, will admit less than a normal amount; and hence the author before alluded to says that congestions of the heart may take place. Even should this not obtain, the blood should not be permitted to become again too stimulating through increased respiration.

Calomel is given in inflammatory diseases because it breaks down the fibrin. May not the veratrum possess a similar property in conjunction with its sedative properties?—is an inquiry which has suggested itself. I think, however, that although this may be to an extent the case, yet it is manifest that its chief action is exerted upon the nervous centres and nervous system.

*Ergot of Wheat.*

Two years since a woman residing eight miles in the country, consulted a physician in this city touching the propriety of superinducing abortion between the third and fourthmonth of utero gestation. This step was not taken by him for very prudential reasons without further consultation, and I was sent for to examine the case. I found her much emaciated from constant and unremitting pain, produced by contractions of the uterus, which resisted effectually the most powerful doses of opium, and other anodyne preparations. There was also great irritability of the stomach, and food was rejected as soon as taken. This state of things had prevailed for some time, and with such constancy, that she was anæmic, and much exhausted. A former pregnancy had been attended during its latter months with similar symptoms, from which, after a hard struggle, she barely recovered, and she felt assured, that as her sufferings were already greater than before, she could not survive this pregnancy, and implored us to bring about abortion.

Another effort however, was made to quiet the irritability of the uterus, but the trial proved unavailing. Deeming the case one in which we were fully authorized to adopt abortive measures, the usual remedies were resorted to for that purpose, closing up, after other experiments, with rupture of the membranes, but the uterus still failed to contract sufficiently to throw off the fœtus until some days had elapsed; at the end of which time, an out-house took fire and burned down in the absence of the male members of the family, during which she became alarmed, left the bed, and hastened out of the house.

The mental emotion arising out of the fright, aroused the uterus to vigorous action, and very soon the fœtus was thrown off, but the placenta remaining and occasioning some uneasiness; the husband requested my attention to the matter, and, accompanied by Dr. J. R. Allen, I visited her. We found the os uteri so completely and resolutely closed, that a manual effort for its removal would have been impossible, as well as impracticable. Ergot, in drachm doses, such too as had been used and pronounced to possess its usual ecbolic properties by others, was repeated, but without producing the desired effect. Whilst there I observed, when running my eye over a field of wheat, that there were on a number of heads, several parasitic grains, similar to, though less than the ergot of rye. The husband, in much anxiety, again reported a failure of the ergot, when I urged upon him the trial of the ergot I saw upon the wheat in his field, which last was not yet ripe for harvest. He returned and having gathered a full table-spoonful of the grains, made a decoction which, in less than half an hour after its exhibition, awakened the contractile powers of the uterine fibre and the placenta was thrown off in a decomposed state, after which the woman recovered rapidly.

There is but little doubt that the ergot used at first, but which failed to produce any effect, was good, and yet in this case it proved incompetent. If this be true, the ergot of wheat was more potent as an ecbolic than that from the rye, because a longer time elapsed between the exhibition of the one than that of the other. Beside this, it is admitted that if ergot act at all, it must do so within half an hour.

At that time I had not seen an account of its use as a substitute for ergot of rye, but some time since I found in the journals that it was used in one or two cases, but when, I do not remember. Comparing date with the case in which it was used by the reporter, I must have used it some time prior to his successful trial of it.

Prof. Henslow found the ergot in the wheat, but makes no mention of its use as a substitute for the ergot of rye, and indeed there are very few who notice it at all. I am well assured that it is not only a substitute for the common ergot, but that it is equal to it, if not superior, in its powers.

I am not aware on what varieties of wheat it is more frequently found; whether it is confined to the winter wheat, (Triticum hybernum,) or the spring variety, (T. æstivum,) I am not prepared to say; nor did it or occur to me at the time above referred to, to make inquiry as to that from which the specimen used was taken. In all cases in which ergot is used in obstetrics, such is the condition of the patient that its effects are required to be prompt. If it failed to act, unhappy results might follow, and hence the necessity of having a pure article and one on which we can rely implicitly. My experience convinces me that great care is required to preserve it for one year, at the end of which time every physician should provide himself with that which is fresh. I have not found any of the preparations reliable, and therefore prefer the mode of preserving the grains closely corked in a bottle with a few grains of camphor.

